# Simulation of a randomly percolated CNT network for an improved analog physical unclonable function

**DOI:** 10.1038/s41598-024-59584-5

**Published:** 2024-04-16

**Authors:** Hyo-In Yang, Hanbin Lee, Jeonghee Ko, Yulim An, Gyeongsu Min, Dong Myong Kim, Dae Hwan Kim, Jong-Ho Bae, Meehyun Lim, Sung-Jin Choi

**Affiliations:** 1 School of Electrical Engineering, Kookmin University, Seoul, 02707 Korea; 2 Mechatronics R&D Center, Samsung Electronics, Gyeonggi-Do, 18448 Korea

**Keywords:** Engineering, Nanoscience and technology

## Abstract

Carbon nanotube networks (CNTs)-based devices are well suited for the physically unclonable function (PUF) due to the inherent randomness of the CNT network, but CNT networks can vary significantly during manufacturing due to various controllable process conditions, which have a significant impact on PUF performance. Therefore, optimization of process conditions is essential to have a PUF with excellent performance. However, because it is time-consuming and costly to fabricate directly under various conditions, we implement randomly formed CNT network using simulation and confirm the variable correlation of the CNT network optimized for PUF performance. At the same time, by implementing an analog PUF through simulation, we present a 2D patterned PUF that has excellent security and can compensate for error occurrence problems. To evaluate the performance of analog PUF, a new evaluation method different from the existing digital PUF is proposed, and the PUF performance is compared according to two process variables, CNT density and metallic CNT ratio, and the correlation with PUF performance is confirmed. This study can serve as a basis for research to produce optimized CNT PUF by applying simulation according to the needs of the process of forming a CNT network.

## Introduction

With the growth of the Internet of Things (IoT), massive amounts of data need to be reproduced, stored, processed, and shared. Additionally, as connectivity to the IoT expands, device identity and encryption techniques have become increasingly important to ensure data security, proper authentication, and secure communication. There are several cryptographic techniques to secure data, but most of these cryptographic primitives use “keys.” Ideally, such primitives should be able to generate random and unique keys and securely store, retrieve, and use these keys as an input into an encryption algorithm to encrypt the data without revealing any information about the keys. However, these tasks are not simple. Previous studies have reported that many security systems have poor key generators, which make them vulnerable to security failures^1,2^. In addition, these keys are usually stored in nonvolatile digital memory, and most IoT products or their constituent elements, such as chips, are vulnerable to various types of security attacks, such as side channel attacks and physical cloning attacks. Therefore, a physically unclonable function (PUF) device has attracted considerable attention to address the aforementioned shortcomings. A PUF in a chip is based on inherent randomness derived from the unavoidable variability that occurs during device fabrication and can be analogous to a fingerprint or an iris of a human. PUF outputs (as the name suggests) cannot be replicated due to their complex physical properties, even if all the parameters in the fabrication process are constant. For example, each instance of PUF receives one or multiple challenges and generates one or multiple responses^3^. The response(s) of each instance will be unique and unpredictable, as the physical phenomenon rendering the response cannot be controlled to produce any fixed type of response. These different types of responses can be associated with different key values and can secure data from external attacks. PUFs can also be used in other applications, e.g., secure RFID systems^4^, IP protection^5^, and device authentication^6^. Several PUF devices have been proposed in the past that exploit electronic^7–9^, optical^10^, and magnetic^11^ properties, etc. There are PUF devices that exploit many other inherent properties of materials. However, the former PUF devices have the disadvantage of relatively low CMOS compatibility, complicated fabrication processes, or large footprint areas.

To address these problems, carbon nanotube (CNT)-based PUFs have been studied and fabricated in recent years^12,13^. Conventional CNTs have been used in various types of CNT network-based thin film transistors (TFTs) due to their numerous advantages, such as excellent electrical properties^14–20^, room-temperature processing compatibility^21^, transparency^22^, and flexibility^23–25^. From a CNT TFT perspective, randomly formed networks and the coexistence of both metallic and semiconducting nanotubes have always been a challenge to solve^26–30^, but this can lead to high reliability from a PUF perspective. During the CNT solution deposition process, CNTs are randomly interconnected with each other to form a network, such that the unique distribution of CNTs within the network is unpredictable and cannot be reproduced identically. A previous study fabricated a PUF device based on solution-processed CNT network TFTs ^31^. This demonstrates that the CNT network itself can be the secret key for high-level hardware security using a simple process with high CMOS compatibility and a small footprint area. However, it is not clear how to control the yield of semiconducting and metallic CNT connections to maximize the randomness of the CNT network.

In this paper, we introduce a random CNT network-based field effect transistor (FET) as a PUF, which exploits the randomness of the CNT network in the channel to generate keys. By presenting the conditions of the CNT network that must be formed through simulation, we maximize the randomness and security level of the CNT network-based PUF. CNT networks can vary significantly during manufacturing due to various controllable process conditions, which have a significant impact on PUF performance. To fabricate a PUF with the best performance, experiments under various conditions are required. However, fabricating such devices has the significant challenge of being very time consuming, expensive, and material intensive. Therefore, by implementing a CNT network according to various variables through simulation, we confirm the correlation between CNT process conditions and PUF performance without direct processing (or without wasted elements) and optimize CNT PUF process conditions. There are many different process methods for forming CNT networks, but in particular, the solution-based CNT network deposition method is completely random because the position or direction of the CNTs present in the solution cannot be controlled during the deposition process. Therefore, this simulation can be applied to any solution-based CNT network deposition method. Additionally, the CNT PUF implemented here is an analog PUF. This analog PUF is distinguished from the multibit digital PUF, such as ternary and quaternary bits, whereas the multibit digital PUF uses multilevel yet discretized values, and the analog PUF uses continuous values^32^. Therefore, our analog PUF can achieve a much higher level of randomness and security compared to traditional digital PUFs of binary or multibit. Since the existing evaluation method of digital PUFs cannot be applied to analog PUFs, we propose a new evaluation method to evaluate the randomness and uniqueness of PUFs. This paper is proposed to be of great significance in fabricating future CNT PUFs with excellent performance.

## Methods

### Monte-Carlo method-based MATLAB simulation

We performed two simulation codes using MATLAB. First, the basic code forms a CNT network with random characteristics^33^. We used a 2D thin film model to reduce the computational demands. We used rand, a random number generation function in MATLAB, to randomly determine the formation position and angle of the CNT lines. The number of CNT lines per area, CNT line length, and m-CNT ratio were set to form random numbers with a normal distribution based on the adjustable variable values. Therefore, it is completely different depending on the number of trials, but as the number of trials increases significantly, numerical results can be obtained using the Monte-Carlo method. Next, the PUF code recognizes the CNT network formed by the base code as a two-terminal device with electrodes on both sides of the x-axis, and extracted the resistance value of the device. Contact was made through the point where the two CNT lines intersected, and a current path was created through the CNTs connected to both terminals. For subsequent node analysis, the resistance of the entire CNT network was calculated by combining Kirchhoff's current law and Ohm's law. Then, this process was repeated *m* × *n* times to obtain *m* × *n* resistance values, which were then extracted into an *m* × *n* matrix.

## Results

**Figure 1:**
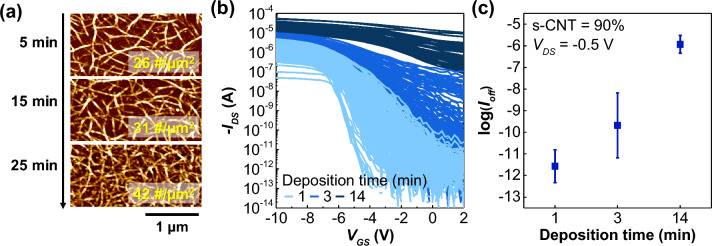
(**a**) AFM images of the CNT network according to deposition time. (**b**) I_DS_-V_GS_ curves of the CNT network transistors. The s-CNT ratio is 90%, and the CNT deposition times are 1, 3, and 14 min, showing differences in electrical properties and process deviation depending on the process conditions. (**c**) Deviation of log(I_off_) according to deposition time. I_off_ is the I_DS_ value at V_GS_ = 0 V.

Figure 1a shows AFM images of the CNT network according to the CNT deposition time. The deposition times are 1, 3, and 14 min, and it can be seen that the density of the CNT network increases as the deposition time increases. Figure 1b shows the drain current (I_DS_)-gate voltage (V_GS_) of the CNT network-based transistor fabricated under the above deposition time conditions. The drain voltage is − 0.5 V, and this is the measurement data for 144 devices for each deposition time^34^. As the deposition time increases, i.e., the density of the CNT network increases, I_on_ defined at V_GS_ = − 10 V and V_D_ = − 0.5 V increases, the possibility of metal interconnection between S/D electrodes also increases, and I_off_ defined at V_GS_ = 0 V and V_D_ = − 0.5 V increases correspondingly. Even if devices are fabricated under the same conditions, their electrical characteristics may vary due to the random nature of the CNT network. Additionally, the degree of deviation varies greatly depending on the process conditions. At this time, the I_off_ value on the log scale showed a large process deviation under the same process conditions, and the deviation also greatly differed according to the deposition time, that is, the density of the CNT network. Figure 1c shows the average and deviation of log(I_off_) according to the deposition time. The average log(I_off_) value differed by density, as did the deviation. When the density of the CNT network is either too low or too high, the deviation is reduced, and the largest process deviation occurs at a specific CNT network density, which is neither too low nor too high. These characteristics can be described in terms of the number of connecting paths between S/D electrodes. At low densities, where the number of connecting paths is too few, process variability is reduced because there are too few possible ways to form connecting paths through which carriers can flow. Even with too many connecting paths, process variability is also reduced due to the averaging effect. Only when the number of connection paths is appropriate is the number of cases in which a path can be formed maximized, and thus, the process deviation has the maximum value. For the off-current region (V_GS_ = 0 V), connecting paths are formed only through the metallic interconnections, such that the CNT network density and the semiconducting/metallic-CNT (s/m-CNT) ratio can control the number of connecting paths. In other words, both the CNT network density and m-CNT ratio are parameters that have a significant impact on the process variation of the CNT network and can be easily controlled. Thus, we evaluated the effects of these two parameters through simulation.

We use MATLAB to conduct the simulations and determine the influence of the density of the CNT network and the m-CNT ratio. This enables quick and easy examination of how physical parameters influence process deviation by obtaining results under various conditions without direct processing. The basic code specifies a region from 0 to the desired value on the x-axis and y-axis and randomly distributes CNTs within this region. The randomly distributed CNTs are expressed as lines, and the rand function is used to make the positions and the rotation angles of the lines have completely random values. (The range of the rand function used to form the position was limited to values within the specified xy area, and to form the rotation angle, it was limited to 0 to 360 degrees.) Other parameters, including the number of CNT lines per area (CNT density), m-CNT ratio, and CNT line length, were set to form random numbers with a normal distribution based on the input value. Next, the PUF code recognizes the CNT network formed in the basic code as a 2-terminal device using both sides of the x-axis as electrodes and extracts the resistance value of the device. The CNTs exhibit different resistance values depending on the type, and the two resistance values are set to have a difference of 4 orders of magnitude, which is the minimum difference compared to other references. The current was allowed to flow only through the path made up of the CNT line connecting the two electrodes. Then, this process is simulated *m* × *n* times to obtain *m* × *n* resistance values, which are then extracted into an *m* × *n* matrix.

**Figure 2:**
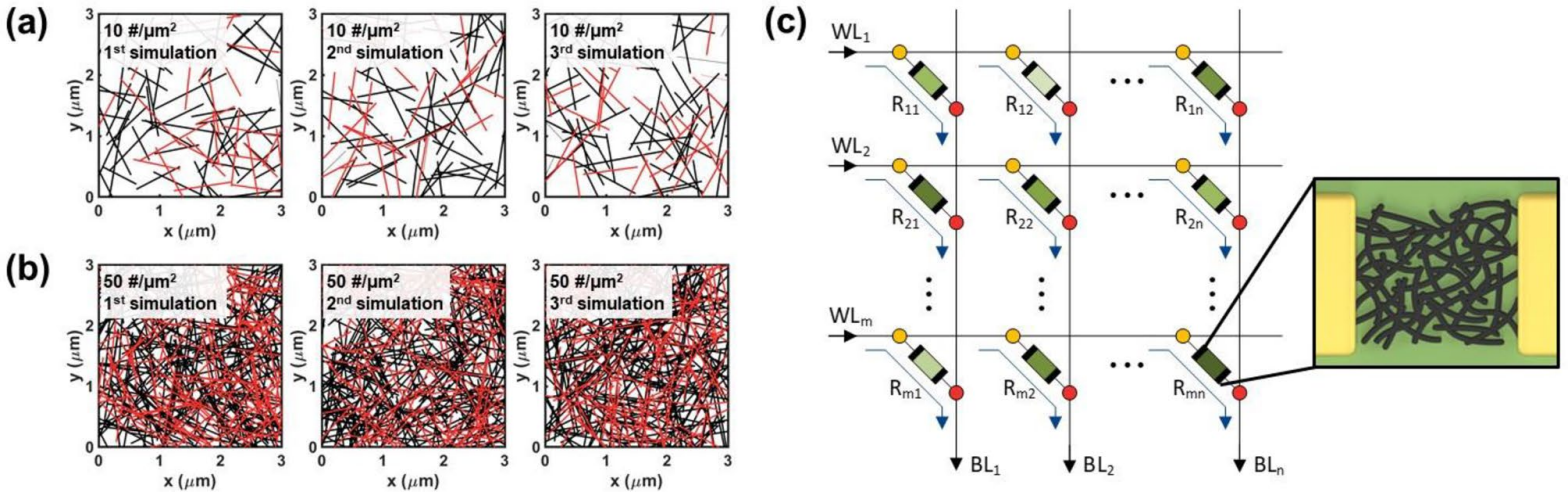
(**a**), (**b**) Results of basic code simulations performed three times each. (**a**) is when the CNT density is 10 #/μm^2^, and (**b**) is when the CNT density is 50 #/μm^2^. (**c**) A circuit schematic diagram of one CNT PUF obtained through the PUF code.

Figure 2a,b show simulation results provided by this basic code. Figure 2a is the result of three simulations when the CNT density is 10 #/µm^2^, and Fig. 2b is the result of three simulations when the CNT density is 50 #/µm^2^. In addition, all other conditions are the same: the CNT length is 1 µm, the m-CNT ratio is 30%, and the network area is 3 μm × 3 μm. Both sides of the x-axis are electrodes, and among the CNT lines, black represents s-CNTs, and red represents m-CNTs. Although the three simulation results in (a) and (b) were all performed under the same conditions, i.e., with the same simulation code, the CNT networks were obtained in completely different shapes by the rand function. Therefore, the shape of the CNT network is unpredictable, and unique shapes can be obtained that vary with each simulation run. Figure 2c shows a circuit schematic diagram of one CNT PUF obtained through the PUF code. Using the PUF code, an *m* × *n* matrix of resistance values is generated, which is regarded as a 2-terminal CNT network device in an *m* × *n* array connected to *m* word lines (WLs) and *n* bit lines (BLs). Therefore, *m*–*n* resistance values for CNT network devices at each position can be obtained, and these can be configured into one PUF. The PUF proposed here is an analog-based PUF that uses analog data, has a much larger capacity for the same area compared to digital bits and has higher security. Additionally, the different resistance values obtained at each location create a unique 2D pattern of the CNT PUF^32^. The CNT PUF can improve encryption by adding matrix information about the location of extracted resistance values, and the matrix can be appropriately arranged to satisfy the needed security level and system specifications.

## Discussion

To compare performance based on two variables, CNT density and m-CNT ratio, the values of the two parameters were adjusted variously in the basic code. The CNT density was divided from 10 #/μm^2^ to 50 #/μm^2^ in 5 #/μm^2^ steps, and the m-CNT ratio was divided into 10%, 20%, 30%, 40%, and 50%. Afterward, a PUF is implemented for each condition through the PUF code, and the performance of the PUF is compared. In this study, 100 simulations were performed using the PUF code to obtain one CNT PUF, and 100 data points obtained from one CNT PUF were arranged in a 10 × 10 matrix. The characteristics of the PUF device are evaluated using the representative PUF parameters, randomness, and uniqueness. Randomness is the diversity of responses to multiple challenges within one PUF. The uniqueness represents the noncorrelation between the responses measured from different chips or arrays. Ideally, the responses of two selected arrays should be uncorrelated, and the logic states of the PUF devices with ideal uniqueness cannot be predicted even if the states of other arrays are known. In the case of digitized PUFs, the comparison between PUFs can be made using binarized data, an array of 1 and 0. For example, randomness is examined by the probability of observing a “1” or “0” in the response of the selected PUF, with an ideal value of 50% for the proportion of the overall random responses. Uniqueness is obtained by counting the number of different responses between two PUFs using the inter-Hamming distance (inter-HD), with an ideal value of 50%. However, since the proposed CNT PUF here uses analog data, another method to evaluate the PUFs should be defined.

First, to evaluate the randomness of the analog data, we normalize the resistance value first. The following function was used to eliminate order differences in resistance values depending on the variable:

1 $$Z={\text{log}}\left(\frac{R}{\mu }\right)+5$$

where *R* is the resistance value, *μ* is the average *R* value within one PUF device, and *Z* is the normalized value. By adding constant value of 5, all data is mapped to the range between 0 and 10, which simplifies calculations. These normalized CNT PUFs can be compared at the same scale and can be displayed in various approaches, such as grid heatmaps and contour maps.

**Figure 3:**
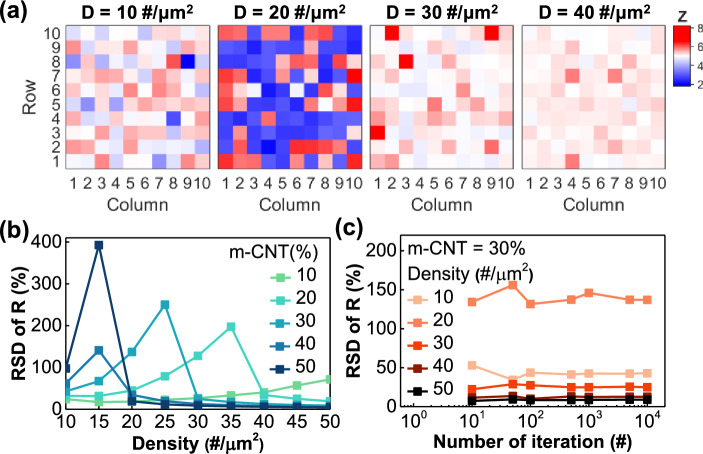
(**a**) Grid heatmap of normalized PUF. From the left, the CNT densities are 10, 20, 30, and 40 #/μm^2^. (**b**) RSD of the resistance value according to the CNT density. Expressed according to the m-CNT ratio of 10, 20, 30, 40, and 50%. (**c**) RSD of the resistance value according to the number of iterations for each CNT density when m-CNT is 30%.

Figure 3a shows the distribution of 10 × 10 array PUFs according to the CNT density as grid heatmaps. From the left, the CNT density is split into 10, 20, 30, and 40 #/μm^2^, and the m-CNT ratio is fixed to 30% in this simulation. In the heatmap, the darker colors indicate deviation from the median, red represents large resistance values, and blue represents low resistance values.

When the CNT density is 10, 30, and 40 #/μm^2^, the color of the PUF is relatively light, which indicates that there is almost no deviation between the data within the PUF. On the other hand, the color of the PUF when the CNT density is 20 #/μm^2^ is relatively dark, and the data deviation within the PUF is severe. Therefore, the randomness within the PUF is expected to be higher at a certain density (CNT density = 20 #/μm^2^) than under conditions where the CNT density is too high or too low. To quantitatively compare randomness, we used the relative standard deviation (RSD) between the data comprising one PUF. RSD is an indicator used to compare the standard deviation of groups with different units. When extracting the resistance value of a CNT device, order differences in the overall distribution of resistance values occur depending on the variable; hence, RSD was applied for comparison according to the variable. This RSD value was calculated by dividing the standard deviation (*S*) of the resistance value by the average value (*µ*) within the PUF.

2 $${\text{RSD}}=\frac{S}{\mu }\times 100\left(\%\right)$$

Figure 3b shows the RSD of the resistance value according to the CNT density for 100 PUFs in a 10 × 10 array and was also classified according to the m-CNT ratio. RSD tends to decrease rapidly at very high or very low densities, peaking at a certain density. As the m-CNT ratio increased, RSD peaked at a lower CNT density. For example, when the m-CNT ratio is 20%, the RSD has the maximum value at a CNT density of 35 #/μm^2^, and when the m-CNT ratio is 50%, the RSD has the maximum value at a CNT density of 15 #/μm^2^. In other words, the number of cases in which a path can be formed increases depending on the proportion of connection paths through which current can flow among the entire CNT network path. As the CNT density increases and the m-CNT ratio increases, the proportion of connection paths increases, so both variables must be considered in the evaluation. To confirm the reliability of the RSD parameter, the RSD values were extracted by increasing the array size of the PUF, i.e., the number of iterations in the simulation. As shown in Fig. 3c, the RSD values for each variable showed constant convergence as the number of iterations increased, thus proving that the RSD parameter was reliable. When the m-CNT ratio is 30%, the RSD appears consistently high at a density of 20 #/μm^2^, and the data show that there is a density point that optimizes the CNT PUF for each m-CNT ratio.

Second, we evaluated the uniqueness of each network. Uniqueness represents the degree of difference between two different PUFs, and this was assessed in two approaches on the analog PUFs in this study. One of them calculates the error factor, and the calculation formula is as follows:

3 $$\text{Error factor}= \sum_{k=1}^{N}\frac{\left|{R}_{i,k}-{R}_{j,k}\right|}{N}\times 100\left(\%\right)$$

where *i* and *j* are two different PUF elements, *k* is the array location within the PUF element, and *N* is the total number of arrays.

**Figure 4:**
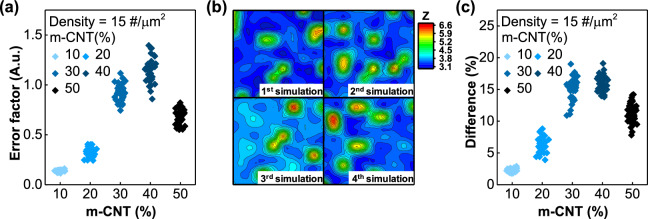
(**a**) Error factor according to the m-CNT ratio. (**b**) Four PUFs simulated under the same conditions are shown as contour map images. (**c**) Difference (%) according to the m-CNT ratio.

Figure 4a shows the error factors calculated for each of the 10 PUF devices in a 10 × 10 array. When the CNT density was 15 #/μm^2^, the m-CNT ratio showed the highest uniqueness at 40%, and it was confirmed that the uniqueness decreased significantly when the m-CNT ratio was too high or too low. We also assessed uniqueness in a more intuitive manner. This includes a method that displays the PUF in the matrix state as a 2D contour map image and then compares the differences between the pictures using an image matching test. To convert the PUF in the matrix state into a 2D pattern image, it passes through the resistance value normalization process mentioned above. Figure 4b shows the PUF image converted to a contour map and is the result of four simulations under the same conditions. Although all four simulation results were obtained under the same conditions, with an m-CNT ratio of 50% and a CNT density of 15 #/μm^2^, completely different shapes were obtained. The contour map conversion process changes the shape of the contour line depending on the relative position of adjacent data values, allowing it to be expressed as a complex fingerprint pattern, further increasing uniqueness. The image match rate for each of these 2D pattern images was calculated using a software program (Prismatic Software Dup Detector v 3.0)^32^. The difference (%) was calculated as the 1 − image match rate (%) and is shown in Fig. 4c. The difference (%) is also highest for an m-CNT ratio = 40% at a CNT density of 15 #/μm^2^. Comparing Fig. 4b,c shows that the uniqueness measured by both methods shares the same tendency. Therefore, both methods are reliable indicators and allow us to find optimal conditions for PUF. Additionally, expressing PUF as a 2D pattern image solves the problem of errors caused by bit inversion by exploiting the relative differences between adjacent resistances instead of using absolute values obtained from electrode pairs.

## Conclusion

We implemented a random CNT network-based analog PUF through simulation. Using the rand function and Monte-Carlo method, a statistical approximation can be obtained by significantly increasing the number of trials while ensuring that the simulated CNT network is completely unpredictable and random for each simulation run on the same code. Using the PUF code, the resistance values of 100 devices obtained by executing the same basic code 100 times are expressed as a PUF of a 10 × 10 array. This array contains matrix position information and can be represented as a unique 2D pattern. The resulting PUF has excellent security because it uses analog data, and since the 2D contour pattern is expressed as the relative difference between adjacent values, it solves the error problem caused by bit inversion. To optimize the CNT solution deposition process conditions most suitable for PUF, the density of the CNTs and the ratio of m-CNTs were varied. The performance evaluation of analog PUFs was provided using a different method from the existing digital PUF performance evaluation parameters. Randomness was compared using the RSD formula, and uniqueness was compared in two ways: calculating the error factor for the resistance value and the image matching test of the 2D contour map. The results confirm that both parameters have an optimal point at a low CNT network density as the m-CNT ratio increases and at a high CNT network density as the m-CNT ratio decreases. After the CNT solution and processing methods are clarified, the corresponding simulation can be used to find the optimal point suitable for PUF. Furthermore, PUF, which is analog in itself, can apply encryption keys in a simple process without an analog-to-digital converter, and is very similar to the encryption method of fingerprints, so it is an encryption technology that will replace fingerprints in the future.

## Data Availability

The data that support the findings of this study are available from the corresponding author upon reasonable request.
